# Hsa_circ_0002483 inhibited the progression and enhanced the Taxol sensitivity of non-small cell lung cancer by targeting miR-182-5p

**DOI:** 10.1038/s41419-019-2180-2

**Published:** 2019-12-16

**Authors:** Xiaoping Li, Bo Yang, Haixia Ren, Ting Xiao, Liang Zhang, Lei Li, Mingjiang Li, Xuhui Wang, Honggang Zhou, Weidong Zhang

**Affiliations:** 1 Department of Thoracic Surgery, Tianjin First Central Hospital, Tianjin, 300192 China; 2 Department of Pharmacy, Tianjin First Central Hospital, Tianjin, 300192 China; 3 College of Pharmacy, Nankai University, State Key Laboratory of Medicinal Chemical Biology, Nankai University, Tianjin, 300350 China; 4 College of Pharmacy, Nankai University, Tianjin Key Laboratory of Molecular Drug Research, Tianjin International Joint Academy of Biomedicine, Tianjin, 300350 China

**Keywords:** RNA, Lung cancer

## Abstract

In this study, we identified a novel circRNA, circ_0002483, and further investigated its functions in the progression and Taxol resistance of NSCLC. We found that circ_0002483 was expressed at low levels in NSCLC tissues and cell lines. Functional assays indicated that circ_0002483 overexpression significantly inhibited NSCLC cell proliferation and invasion in vitro and in vivo and enhanced the sensitivity of NSCLC cells to Taxol. Mechanistically, circ_0002483 was identified to sponge multiple miRNAs including miR-182-5p (also named miR-182), miR-520q-3p, miR-582-3p, miR-587, and miR-655. In addition, circ_0002483 was also demonstrated to regulate the expression of GRB2, FOXO1, and FOXO3, three target genes of miR-182-5p, by sponging miR-182-5p. Circ_0002483 was demonstrated to inhibit NSCLC progression in vitro and in vivo and enhanced the sensitivity of NSCLC cells to Taxol by sponging miR-182-5p to release the inhibition on GRB2, FOXO1, and FOXO3 mRNAs.

## Introduction

According to the annual estimate by the American Cancer Society for 2019, there will be ~1,762,450 new cancer cases and 606,880 cancer deaths projected to occur in the United States, among which lung cancer remains one of the most common cancers both in terms of incidence and mortality^1^. Non-small cell lung cancer (NSCLC), mainly including adenocarcinoma, squamous cell carcinoma, and large cell carcinoma, accounts for more than 80% of all lung cancers^2,3^. Due to the lack of effective diagnostic measures and biomarkers, most NSCLC patients are diagnosed at the advanced stage IIIB or IV, which is characterized by distant metastasis and poor prognosis^4,5^. Chemotherapy has proven survival benefits for patients with advanced, unresectable NSCLC; however, the overall 2-year survival rate for chemotherapy-treated NSCLC patients remains <20%^6^. The combination of radiotherapy and chemotherapy is currently used as the standard therapeutic strategy for advanced NSCLC patients without pleural or pericardial effusion^7^. Tumor cell metastasis is considered to be the leading cause of NSCLC-associated deaths in clinical therapy, and so far, there have been no effective measures to suppress NSCLC cell metastasis^8,9^.

Taxol (generic name paclitaxel), belonging to a group of diterpenoid alkaloids with anti-tumor capacity, is an antimicrotubule agent that targets taxane binding sites and subsequently blocks depolymerization, inhibiting cell proliferation^10^. Taxol is widely used as a first-line chemotherapy agent in the treatment of multiple types of tumors, including NSCLC^10,11^. Currently, Taxol-based combination therapy with cisplatin or carboplatin is a preferred choice for advanced NSCLC patients with metastatic activity^12^. However, the anti-tumor activity of Taxol is frequently limited by various factors, such as overexpression of β-tubulin isoforms, mitotic checkpoint alterations, and ATP-binding cassette transporters, resulting in primary or secondary resistance to Taxol and ultimately inducing local recurrence and distant metastasis^13^. Therefore, it is essential to investigate the pathogenesis of NSCLC and understand the molecular mechanisms of Taxol resistance.

It is well known that only ~2% of the human genome has protein-encoding capacity, and most of the rest of human transcripts are termed noncoding RNAs (ncRNAs) without the capacity to encode protein^14^. MicroRNAs (miRNAs), long noncoding RNAs (lncRNAs), and circular RNAs (circRNAs) are the three main types of ncRNAs^15^. Previous studies have shown that the dysregulation of miRNAs, lncRNAs, and circRNAs is involved in the tumorigenesis of multiple human cancers by regulating corresponding oncogenes or tumor suppressor genes^16,17^. MiRNAs and lncRNAs have been reported to be associated with the Taxol resistance in diverse human cancers, including breast cancer, liver cancer and lung cancer^18,19^. However, the roles of circRNA in the Taxol resistance in lung cancer remain largely undetermined. Recently, Ning Xu et al. reported the profiles of differentially expressed circRNAs in Taxol-resistant NSCLC, showing 2909 significantly increased and 8372 decreased circRNAs in Taxol-resistant NSCLC cells compared with normal NSCLC cells^20^. In the present study, we identified a novel circRNA (circ_0002483) in the top 20 downregulated circRNAs reported by Ning Xu et al. and investigated the effects of circ_0002483 on the progression and Taxol resistance of NSCLC.

## Materials and methods

### NSCLC tissue samples and cell lines

NSCLC tissue samples and adjacent normal tissue samples were collected from patients who were diagnosed with NSCLC in Tianjin First Central Hospital during 2010–2018. Written informed consent was obtained from all NSCLC patients, and this study was approved by the ethics committee of Tianjin First Central Hospital. The normal lung epithelial cell line (HBE), four NSCLC cell lines (A549, H1299, H358, and PC9) and two Taxol-resistant NSCLC cell lines (A549/Taxol, H1299/Taxol) were all obtained from the Type Culture Collection of Chinese Academy of Sciences (Shanghai, China). The cells were cultured at 37 °C in RPMI-1640 medium (HyClone Laboratories Inc., USA) supplemented with 10% fetal bovine serum (FBS) and 1% penicillin/streptomycin at 5% CO_2_ and 95% air.

### Transfections of siRNAs

Negative control (NC) and siRNAs were obtained from GenePharma (Shanghai, China). The sequence of hsa_circ_0002483 siRNAs is 5′-AACAGAATATGACAGATACCT-3′. A549 (5 × 10^4^ cells/well) were seeded in 6-well plates and transfected with NC and siRNAs for 48 h using Lipofectamine 3000 Reagent (Life Technologies, Cat. #L3000015).

### Plasmid construction and cell transfection

To overexpress hsa_circ_0002483, PrimerSTAR Max DNA Polymerase Mix (Takara) was applied to amplify the full-length cDNA of hsa_circ_0002483 from 293T cells, and the obtained cDNAs were inserted into the overexpression vector pLCDH-ciR (GenePharma, Shanghai, China), which has a front and back circular frame. Hsa_circ_0002483 was verified by direct sequencing. A549 and H1299 cells (1 × 10^5^ cells/well) were seeded in 6-well plates and transfected with the hsa_circ_0002483-expression vector or empty vector using Lipofectamine 3000 Reagent (Life Technologies, Cat. #L3000015) according to the manufacturer’s instructions.

### RNA extraction and quantitative real-time PCR (RT-PCR) assay

Total RNA from NSCLC tissues and cell lines was extracted using TRIzol reagent (Invitrogen, Carlsbad, CA, USA), and RNA quality was determined by NanoDrop 2000c (Thermo Scientific, Waltham, USA). Then, the Bestar qPCR RT Kit (#2220, DBI Bioscience, China) was used to produce cDNA from 2 μg of total RNA. RT-PCR was performed on the ABI7500 system with Bestar qPCR MasterMix (#2043, DBI Bioscience, China). The sequence of primers used in the present study is shown in Table 1. The expression of circ_0002483, GRB2, FOXO1, and FOXO3 was normalized to GAPDH, miR-182-5p expression was normalized to U6, and gene expression was quantified via the 2^−ΔΔCt^ method.

**Table 1:** The sequences of primers in this study.

Gene	Primer sequences
GAPDH	Forward: 5′-TATGATGATATCAAGAGGGTAGT-3′ Reverse: 5′-TGTATCCAAACTCATTGTCATAC-3′
has_circ_0002483	Forward: 5′-TGCCAAAAGGATTTCTAAACCAGT-3′ Reverse: 5′-TTGGGGTCAAGGTAAGCAGC-3′
PTK2	Forward: 5′-TGGGCGGAAAGAAATCCTGC-3′ Reverse: 5′-GGCTTGACACCCTCGTTGTA-3′
GRB2	Forward: 5′- ATTCCTGCGGGACATAGAACA-3′ Reverse: 5′- GGTGACATAATTGCGGGGAAAC-3′
FOXO1	Forward: 5′-CCCAGGCCGGAGTTTAACC-3′ Reverse: 5′-GTTGCTCATAAAGTCGGTGCT-3′
FOXO3	Forward: 5′-CCCTCTCGGACTCTCTCTCA-3′ Reverse: 5′-AAATCCAACCCATCAGCATC-3′
miR-182-5p	Forward: 5′-GTCGTATCCAGTGCGTGTCGTGGAGTC-3′ Reverse: 5′-GGCAATTGCACTGGATACGACAGTGTG-3′
U6	Forward: 5′-CGCTTCGGCAGCACATATACTAA-3′ Reverse: 5′-GCTGTCAACGATACGCTACCTA-3′

### Western blot assay

Total protein was extracted from the treated A549 and H1299 cells using RIPA Lysis Buffer (Vazyme, cat. no. FD008). And the concentration was then quantitated using Pierce BCA protein assay kit (Rockford). The equivalent proteins in each group were isolated using 10% SDS-PAGE according to their molecular weights, and the separate proteins were transferred to PVDF membrane (Millipore). After blocking with 5% skim milk for 2 h, the membranes were incubated with primary antibodies at 4 °C overnight, followed by second antibody (Abcam) for 1 h. Finally, the membranes were treated with Enhanced ECL luminescence detection kit (Vazyme; E411-04), and the results were examined on FluorChem™ M System. The primary antibody included anti-FOXO3 (Abcam, ab17026), anti-FOXO1 (Abcam, ab39670), anti-GRB2 (Abcam, ab111031), and anti-GAPDH (Abcam, ab37168).

### Cell Counting Kit-8 (CCK-8) assay

NSCLC cells were trypsinized and seeded into 96-well plates at a concentration of 2 × 10^4^ cells/ml. After culture at 37 °C for 24 h, NSCLC cells were transfected with the corresponding oligonucleotides (Circ OE, si-Circ, and anti-miR-182-5p), followed by treatment with various concentrations of Taxol (0, 100, 200, 300, 400, and 500 nM) and were incubated at 37 °C for 72 h. Subsequently, CCK-8 solution (10 µl, Beyotime Institute of Biotechnology, Shanghai, China) was added to 100 µl of culture medium containing 10% FBS. The absorbance values were measured at 450 nm.

### Colony formation assay

Briefly, treated NSCLC cells were seeded into 6-well plates at a density of 1000 cells/well. After incubation at 37 °C at 5% CO_2_ and 95% air for 2 weeks, the colonies were fixed and stained with crystal solution, and the number of colonies was calculated. The colony formation rate was calculated and normalized to the control group.

### Self-renewing spheroid formation assay

NSCLC cells (500 cells/well) were seeded into 6-well plates and cultured in serum-free RPMI-1640 medium containing EGF (20 ng/ml, BD Biosciences), B27 (1:50, Invitrogen), and insulin (4 mg/ml, Sigma) for 2 weeks. Then, the number of NSCLC cell spheres (tight, spherical, non-adherent masses > 50 µm in diameter) was imaged and counted.

### Invasion analysis

Transwell chambers (8-µm pores, Corning Incorporated, USA) with Matrigel matrix (BD Biosciences, USA) were used to examine the invasive ability of NSCLC cells. In brief, treated NSCLC cells were harvested and resuspended in serum-free culture medium at a final concentration of 1 × 10^5^ cells/ml. Then, 200 µl NSCLC cell suspension was added to the upper chamber, and 500 µl culture medium containing 10% FBS was added to the lower chamber. After 24 h incubation at 37 °C, the invaded NSCLC cells were fixed and stained with 0.5% crystal violet (Beyotime Institute of Biotechnology, China).

### In vivo tumor growth assay

In vivo tumor growth assays were performed using male BALB/c mice (5–8 weeks old) provided by Tianjin First Central Hospital to evaluate the effects of circ_0002483 overexpression on NSCLC progression. Treatment of the nude mice was approved by the Institutional Animal Care and Use Committee of the Tianjin First Central Hospital. In brief, A549 cells stably transfected with empty vector or Circ OE were harvested and resuspended in culture medium (1 × 10^5^ cells/ml). Then, 200 μL A549 cell suspensions were subcutaneously injected into the left flank of nude mice. Tumor volume was measured every 3 days until 21 days after injection. Tumor volumes were measured as the length × width^2^ × 0.5.

### Dual-luciferase reporter assay

To confirm the interaction between circ_0002483 and miR-182-5p, the wild-type (WT) and mutant (Mut) fragments of circ_0002483 containing putative miR-182-5p binding sites were amplified and subcloned into the pGL3 (Promega, USA) vector to form circ_0002483-WT and circ_0002483-Mut recombinant plasmids. A549 and H1299 cells were seeded into 96-well plates at a concentration of 1 × 10^4^ cells/well and cultured at 37 °C overnight. A549 cells were cotransfected with miR-182-5p mimics and circ_0002483-WT or circ_0002483-Mut, and H11299 cells were cotransfected with anti-miR-182-5p and circ_0002483-WT or circ_0002483-Mut. Then, the firefly and Renilla luciferase activities of treated A549 and H1299 cells were detected using the Dual-Luciferase Assay System (Promega), and Renilla luciferase activity was normalized to firefly luciferase activity. The interaction between circ_0002483 and miR-520q-3p, miR-582-3p, miR-587 or miR-655 was also verified to be the same as that between miR-182-5p and circ_0002483.

### RNA immunoprecipitation (RIP) assay

The biotin-labeled miR-182-5p probe was utilized to validate the interaction between miR-182-5p and circ_0002483 in H1299 and A549 cells. The probe was designed and obtained from Sangon Biotech (Shanghai, China). H1299 and A549 cells were lysed with RIP lysis buffer and incubated with magnetic beads overnight at 4 °C. The enrichment of circ_0002483 was analyzed by RT-qPCR assay.

### Kyoto Encyclopedia of Genes and Genomes (KEGG) analysis

Putative target mRNAs of miR-182 were predicted through TargetScan (http://www.targetscan.org/vert_71/). Biological pathways of the miRNA target genes were analyzed by DAVID (https://david.ncifcrf.gov/) and by Rx64 version 3.5.1.

### Statistical analysis

Data are expressed as the mean ± SD. One-way analysis of variance was conducted using GraphPad (Prism 7, GraphPad Prism Software, La Jolla, CA, USA) and was applied to analyze the differences between the groups. A *P* value <0.05 was considered significant.

## Results

### Decreased circ_0002483 was found to be correlated with a poor prognosis in NSCLC

**Fig. 1:**
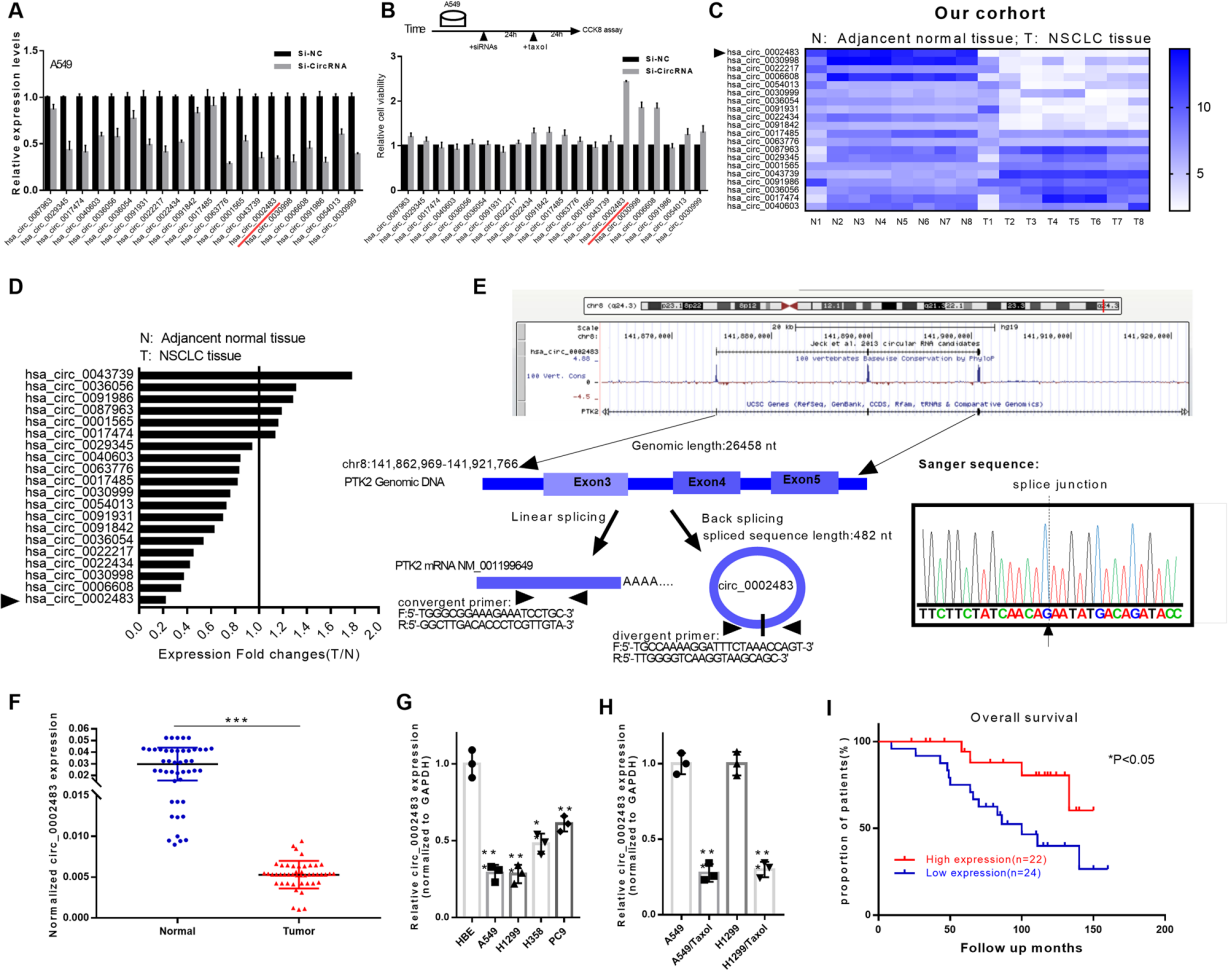
Decreased circ_0002483 expression was found to be correlated with a poor prognosis of NSCLC patients. According to the circRNA expression profiles of Taxol-resistant NSCLC reported in a previous study, the top 20 downregulated circRNAs were selected for our study. After individually silencing the expression of the 20 circRNAs in A549 cells, the cells were treated with Taxol (10 nM), and **a** the transfection effects of siRNAs were verified by RT-qPCR and **b** CCK-8 assay was performed to examine the cell viability of the treated A549 cells. **c**, **d** Expression of the 20 circRNAs was detected in 8 pairs of NSCLC and normal tissues by RT-qPCR. **e** A schematic diagram of the genomic locations of circ_0002483 and circ_0002483, which was validated by RT-PCR using divergent primers and Sanger sequencing. **f** Relative circ_0002483 expression in 46 pairs of NSCLC and adjacent normal tissues was measured via RT-qPCR assay, ***P < 0.001. **g** RT-qPCR analysis of circ_0002483 in one normal lung epithelial cell line (HBE) and four NSCLC cell lines (A549, H1299, H358, and PC9), **P < 0.01, ***P < 0.001. **h** Relative circ_0002483 expression was examined through RT-qPCR in A549, A549/Taxol, H1299, and H1299/Taxol cells, ***P < 0.001. **i** The survival rate of NSCLC patients with high or low circ_0002483 expression was analyzed by Kaplan–Meier survival plots, *P < 0.05.

Ning Xu et al. reported a circRNA expression profile in Taxol-resistant NSCLC obtained through bioinformatics methods that showed the top 20 upregulated and downregulated circRNAs^20^. To further evaluate the biological functions of specific circRNAs in NSCLC, we knocked down the expression of the top 20 downregulated circRNAs individually, followed by treatment with Taxol and then RT-qPCR analysis of the transfection effects or a CCK-8 analysis of cell viability (Fig. 1a, b upper panel). The results of the RT-qPCR assay demonstrated the transfection effects of the top 20 circRNAs and showed that the expression of most circRNAs was significantly decreased after transfection with the siRNAs (Fig. 1a).

The results of the CCK-8 assay indicated that the viability of the circ_0002483 siRNA-treated group exhibited the most obvious change (Fig. 1b lower panel). Moreover, we analyzed the top 20 downregulated circRNAs in 8 pairs of NSCLC and adjacent normal tissue samples, and circ_0002483 also showed the most obvious change (Fig. 1c, d). Circ_0002483 is located at chr8:141862969-141921766, which was confirmed by sanger sequencing of the RT-PCR products amplified via specific divergent primers (Fig. 1e). Next, we found that circ_0002483 was significantly downregulated in NSCLC tissue samples compared with normal tissue samples (*n* = 46, Fig. 1f). In addition, compared with that in HBE cell lines, circ_0002483 expression was significantly decreased in A549, H1299, H358, and PC9 cells (Fig. 1g) and was downregulated in A549/Taxol and H1299/Taxol compared with the parental cell lines A549 and H1299 cells (Fig. 1h). In addition, NSCLC patients with low circ_0002483 expression exhibited a worse prognosis than those with high circ_0002483 expression (Fig. 1i).

### Overexpression of circ_0002483 inhibited NSCLC cell proliferation and invasion in vitro and in vivo

**Fig. 2:**
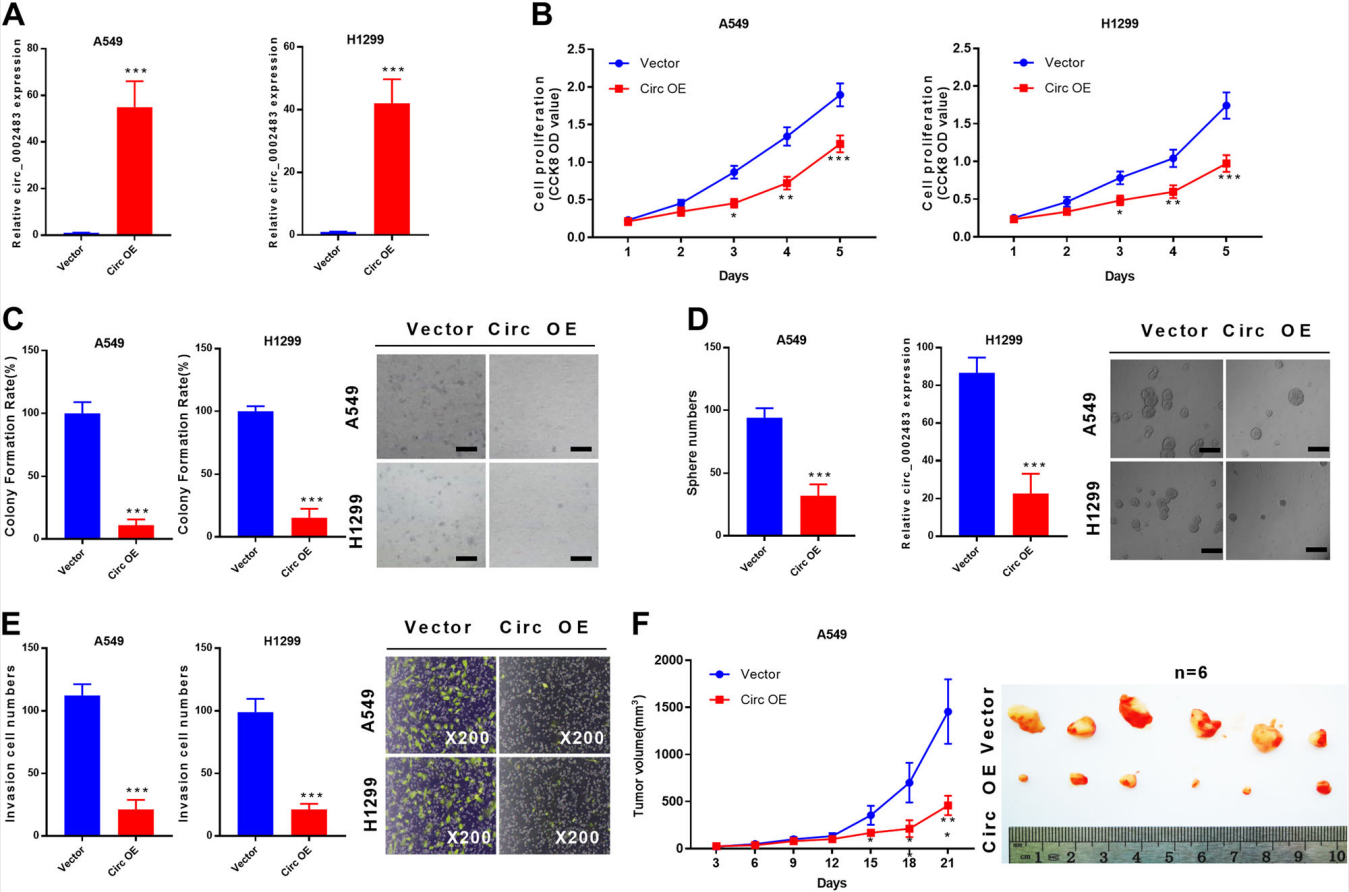
Overexpression of circ_0002483 inhibited NSCLC cell proliferation and invasion in vitro and in vivo. **a** The overexpression efficiency of Circ OE in A549 and H1299 cells was determined by RT-qPCR, ***P < 0.001 vs vector. **b**, **c** The effects of circ_0002483 overexpression on cell proliferation were evaluated by CCK-8 assay and colony formation assay in A549 and H1299 cells, respectively, ***P < 0.001 vs vector. **d** A self-renewing spheroid formation assay was performed in A549 and H1299 cells transfected with vector or Circ OE to evaluate the effects of circ_0002483 on tumor sphere formation, ***P < 0.001 vs vector. **e** The invasive ability of A549 and H1299 cells transfected with vector or Circ OE was assessed by Transwell assay, ***P < 0.001 vs vector. **f** An in vivo tumor growth assay was carried out to further study the effects of circ_0002483 overexpression on NSCLC progression, ***P < 0.001 vs vector.

To investigate the biological functions of circ_0002483 in NSCLC, we overexpressed circ_0002483 by transfecting A549 and H1299 cells with Circ_0002483 (Circ OE) (Fig. 2a). The CCK-8 assay and colony formation assay showed that circ_0002483 overexpression significantly suppressed cell viability in both A549 and H1299 cells compared with the vector group (Fig. 2b, c). The self-renewing spheroid formation assay showed that Circ OE treatment resulted in a significant downregulation of sphere number in A549 and H1299 cells compared with vector treatment (Fig. 2d). Moreover, the Transwell assay indicated that the numbers of invasive A549 and H1299 cells transfected with Circ OE were significantly decreased compared with those cells transfected with the vector (Fig. 2e). To further evaluate the effects of circ_0002483 on NSCLC tumorigenesis, an in vivo xenograft tumor formation assay indicated that the average tumor volume in the Circ OE group was obviously smaller than that in the empty vector group (Fig. 2f).

### Overexpression of circ_0002483 enhanced the sensitivity of NSCLC cells to Taxol

**Fig. 3:**
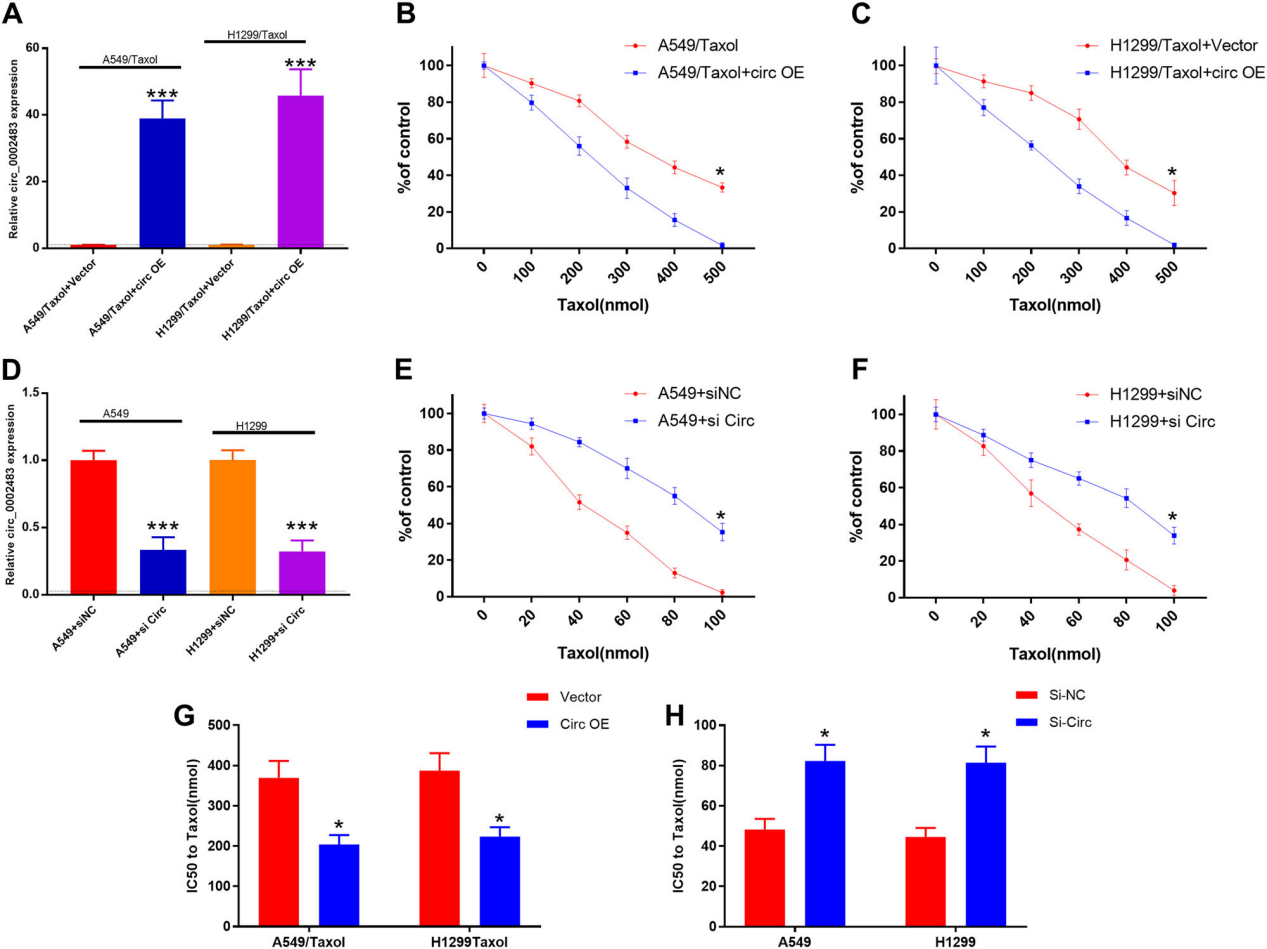
Overexpression of circ_0002483 enhanced the sensitivity of NSCLC cells to Taxol. **a** RT-qPCR analysis of circ_0002483 in A549/Taxol and H1299/Taxol cells treated with vector or Circ OE, ***P < 0.001. **b**, **c** A549/Taxol and H1299/Taxol cells transfected with vector or Circ OE were analyzed by CCK-8 assay following a 48-h treatment with Taxol (0, 100, 200, 300, 400, or 500 nM), *P < 0.05. **d** RT-qPCR analysis of circ_0002483 of A549/Taxol and H1299/Taxol cells treated with si-NC or si-Circ, ***P < 0.001. **e**, **f** A549/Taxol and H1299/Taxol cells stably transfected with si-NC or si-Circ were analyzed by CCK-8 assay following a 48-h treatment with Taxol (0, 100, 200, 300, 400, or 500 nM), *P < 0.05. **g**, **h** The IC50 values of A549/Taxol and H1299/Taxol transfected with Circ OE or si-Circ were determined by CCK-8 assay, *P < 0.05. OE, overexpression; si-NC, negative control siRNAs; si-Circ, circ_0002483 siRNAs.

Our previous data showed that circ_0002483 was significantly downregulated in Taxol-resistant NSCLC cells, implying that targeting circ_0002483 would overcome Taxol resistance. The overexpression efficiency of Circ OE in A549/Taxol and H1299/Taxol cells was determined by RT-qPCR (Fig. 3a), and the knockdown efficiency of si-Circ was also determined via RT-qPCR (Fig. 3d). The CCK-8 assay showed that circ_0002483 overexpression significantly enhanced the sensitivity of A549/Taxol and H1299/Taxol cells to Taxol (Fig. 3b, c), while circ_0002483 knockdown remarkably attenuated the sensitivity of A549/Taxol and H1299/Taxol cells to Taxol (Fig. 3e, f). Moreover, we found that Circ OE treatment resulted in a significant downregulation of the IC50 value of A549/Taxol and H1299/Taxol cells (Fig. 3g), while si-Circ treatment exhibited the opposite effect on the IC50 value of A549/Taxol and H1299/Taxol cells (Fig. 3h).

### Circ_0002483 functions as a sponge for multiple miRNAs in NSCLC cells

**Fig. 4:**
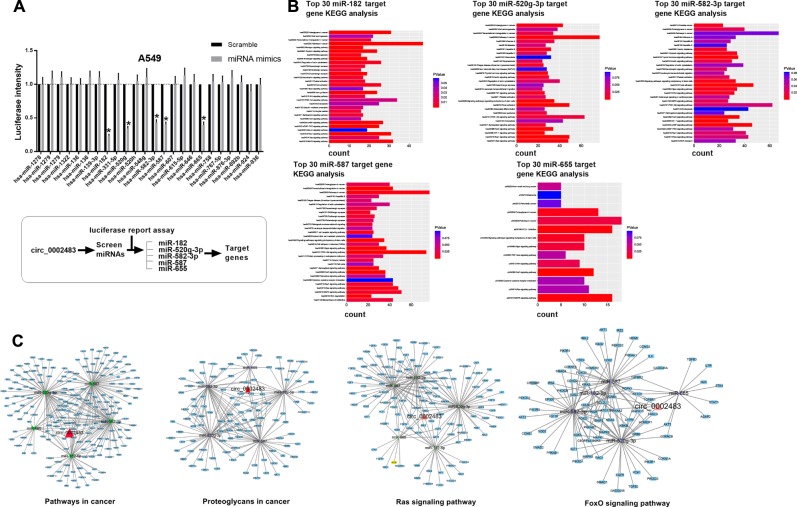
Circ_0002483 sponged multiple miRNAs in NSCLC cells. **a** Upper panel, dual-luciferase reporter assay was performed to verify the interaction between predicted miRNAs and circ_0002483 in A549 cells. Lower panel, schematic diagram a screen for target genes of circ_0002483, *P < 0.05. **b** KEGG pathway analysis of five miRNAs targeted by circ_0002483. **c** Four critical pathways that have been identified in the KEGG pathway analysis of all five target miRNAs of circ_0002483.

By using bioinformatics analysis, we found that a total of 24 miRNAs were identified as potential targets of circ_0002483 in NSCLC. A dual-luciferase reporter assay was performed to further confirm the interaction between circ_0002483 and its predicted miRNAs in A549 cells. Among the 24 predicted miRNAs, only five miRNAs (miR-182-5p, miR-520q-3p, miR-582-3p, miR-587, and miR-655) significantly attenuated the luciferase activity of A549 cells (Fig. 4a). We subsequently predicted the target genes of miR-182-5p, miR-520q-3p, miR-582-3p, miR-587, and miR-655 by using TargetScan 7.2 and analyzed the identified target genes in a KEGG pathway analysis (Fig. 4b). Based on this analysis, the top 30 target genes of the five miRNAs were found to be involved in the following four common, critical pathways: pathways in cancer, proteoglycans in cancer, Ras signaling pathway and FoxO signaling pathway (Fig. 4c).

### Circ_0002483 directly bound to and was negatively correlated with miR-182-5p

**Fig. 5:**
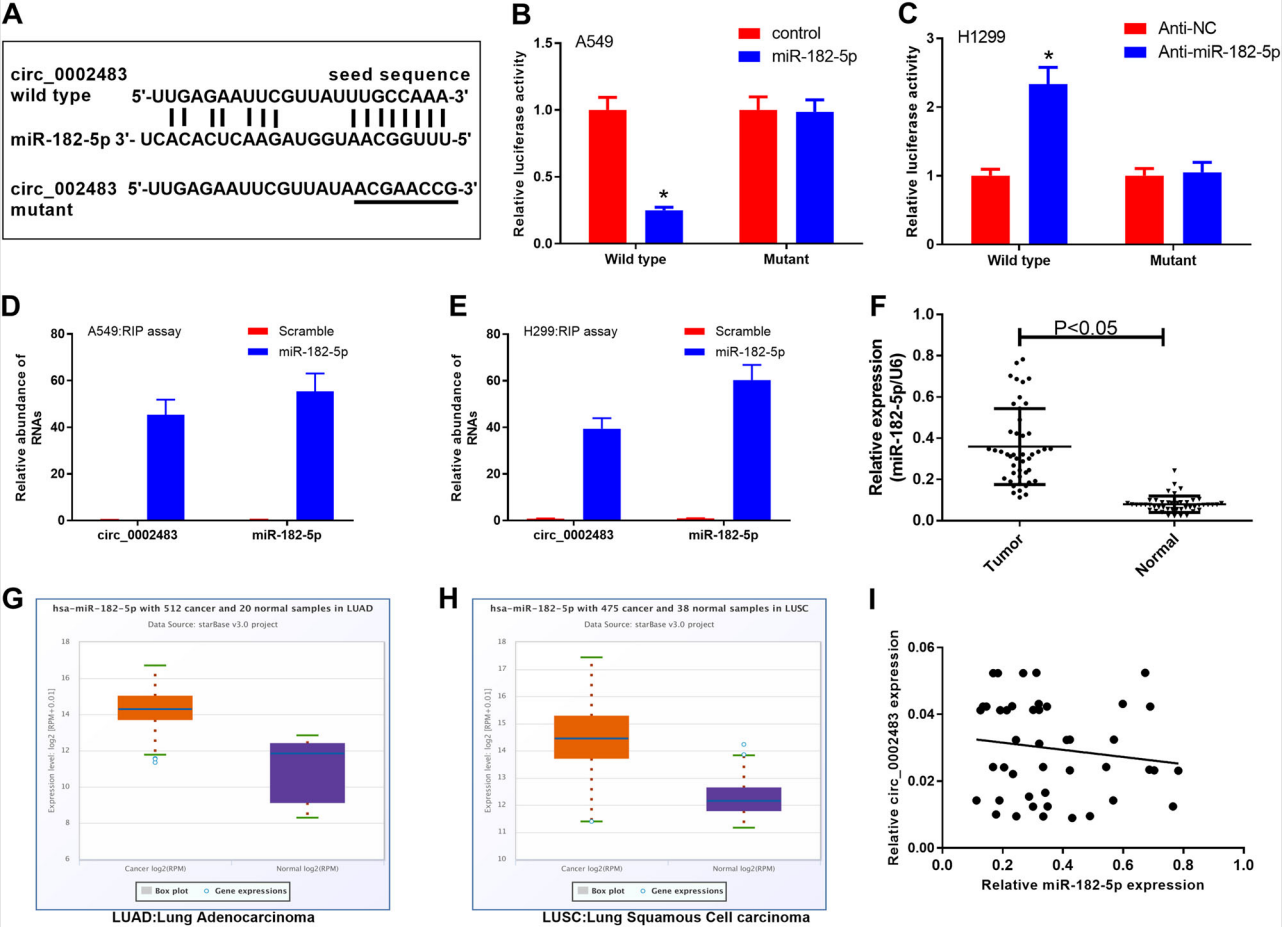
Circ_0002483 directly bound to and showed a negative correlation with miR-182-5p. **a** The putative binding sites between miR-182-5p and circ_0002483. **b**, **c** Interaction between miR-182-5p and circ_0002483 was verified via dual-luciferase reporter assay in A549 and H1299 cells, *P < 0.05. **d**, **e** RIP assay was carried out to determine the interaction between miR-182-5p and circ_0002483 in A549 and H1299 cells. **f** Relative miR-182-5p expression was detected in NSCLC and adjacent normal tissues through RT-qPCR assay. **g** Expression of miR-182 in 512 cancer and 20 normal samples in lung adenocarcinoma (LUAD). **h** Expression of miR-182 in 475 cancer and 38 normal samples in lung squamous cell carcinoma (LUSC). **i** Correlation between miR-182-5p and circ_0002483 in NSCLC.

MiR-182-5p was selected for further study because it showed the greatest fold change in the previous luciferase report assay. The sequence of the putative binding sites between miR-182-5p and circ_0002483 is shown (Fig. 5a). The dual-luciferase reporter assay indicated that miR-182-5p could significantly attenuate the luciferase activity of A549 and H1299 cells driven by the wild-type circ_0002483 recombinant plasmid compared with that of the control group and was rescued by the mutant circ_0002483 recombinant plasmid (Fig. 5b, c). The RIP assay showed a specific enrichment of circ_0002483 and miR-182-5p in the miR-182-5p probe group compared with the scramble group in both A549 and H1299 cells (Fig. 5d, e). In addition, the expression level of miR-182-5p in NSCLC tissues was significantly higher than in adjacent normal tissues (Fig. 5f). Moreover, TCGA Data Portal results from starBase v3.0 also showed that miR-182-5p was significantly upregulated in cancer tissues compared with their normal counterparts in lung adenocarcinoma (LUAD) and lung squamous cell carcinoma (LUSC) (Fig. 5g, h). Furthermore, the expression of miR-182-5p and circ_0002483 were negatively correlated in NSCLC (*r* = −0.1401) but the difference was not statistically significant (*P* = 0.3531) (Fig. 5i).

### Knockdown of miR-182-5p inhibited NSCLC cell proliferation and invasion and enhanced the sensitivity of NSCLC cells to Taxol

**Fig. 6:**
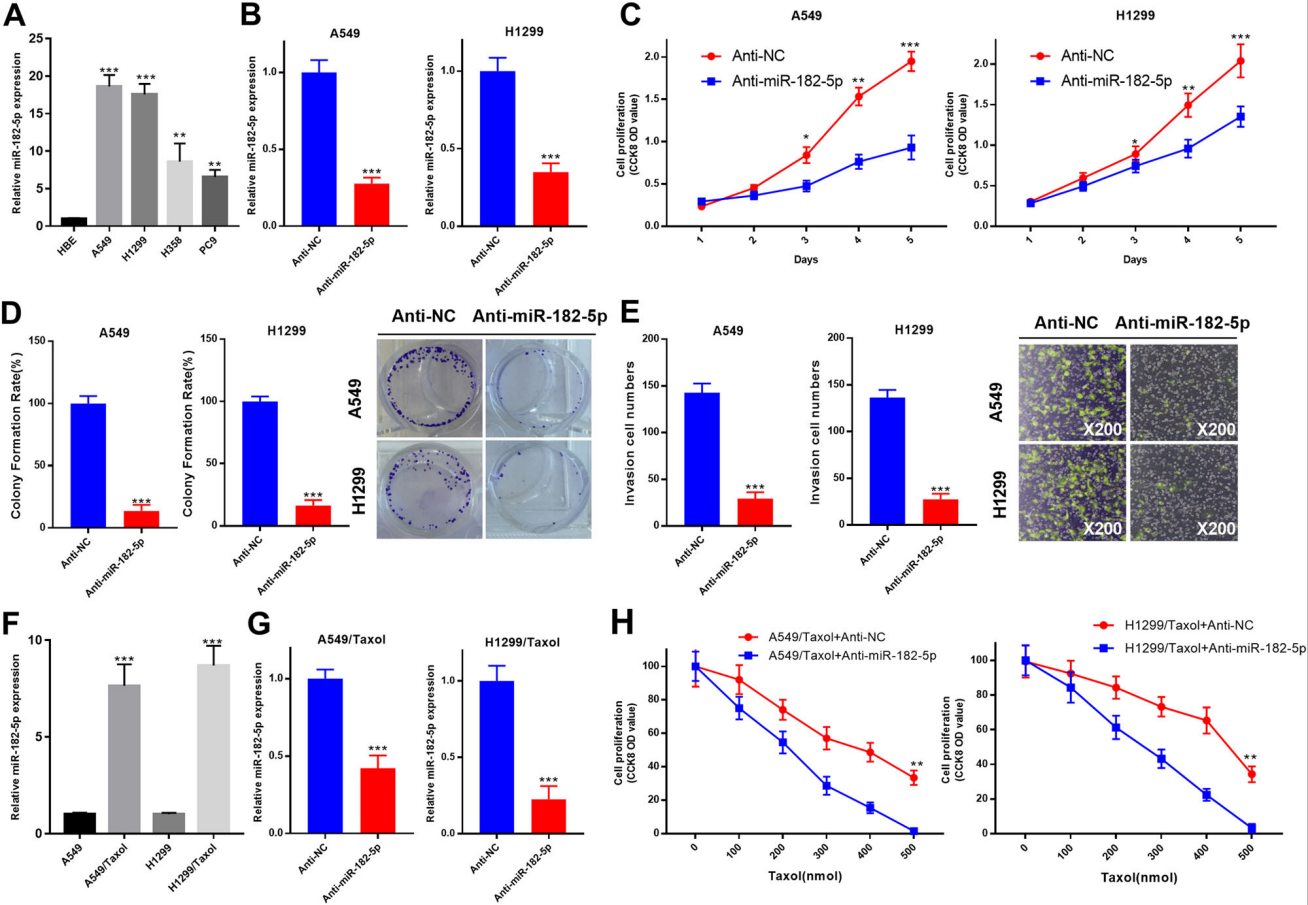
Knockdown of miR-182-5p inhibited NSCLC cell proliferation and invasion and enhanced the sensitivity of NSCLC cells to Taxol. **a** Relative miR-182-5p expression was measured in HBE, A549, H1299, H358 and PC9 cells by RT-qPCR assay, **P < 0.01, ***P < 0.001. **b** After treating A549 and H1299 cells with anti-NC or anti-miR-182-5p, relative miR-182-5p expression was measured by RT-qPCR assay, ***P < 0.001. **c** CCK-8 analysis of A549 and H1299 cells transfected with anti-NC or anti-miR-182-5p, ***P < 0.001. **d** The effects of miR-182-5p knockdown on cell proliferation were evaluated by using a colony formation assay, ***P < 0.001. **e** Transwell assays were utilized to assess the invasive ability of A549 and H1299 cells treated with anti-NC or anti-miR-182-5p, ***P < 0.001. **f** Relative miR-182-5p expression in A549 and H1299, as well as their Taxol-resistant derivatives A549/Taxol and H1299/Taxol, was measured via RT-qPCR assay, ***P < 0.001. **g** The knockdown efficiency of anti-miR-182-5p was determined by RT-qPCR in A549/Taxol and H1299/Taxol cells, ***P < 0.001. **h** A549/Taxol and H1299/Taxol cells stably transfected with anti-NC or anti-miR-182-5p were analyzed by CCK-8 assay following a 48-h treatment with Taxol (0, 100, 200, 300, 400, or 500 nM), **P < 0.01.

Compared with that in the HBE cell line, miR-182-5p was significantly upregulated in the NSCLC cell lines A549, H1299, H358, and PC9 (Fig. 6a). Then, we knocked down the expression of miR-182-5p in A549 and H1299 cells with miR-182-5p inhibitors (anti-miR-182-5p), and the knockdown efficiency was examined (Fig. 6b). Subsequently, CCK-8, colony formation and Transwell assays in A549 and H1299 cells indicated that anti-miR-182-5p significantly inhibited cell proliferation and invasion (Fig. 6c–e). In addition, we found that miR-182-5p was significantly higher in A549/Taxol and H1299/Taxol cells than in A549 and H1299 cells (Fig. 6f). Anti-miR-182-5p in A549/Taxol and H1299/Taxol cells effectively decreased its expression (Fig. 6g). Blocking miR-182-5p significantly enhanced the toxicity of Taxol to A549/Taxol and H1299/Taxol cells (Fig. 6h).

### GRB2, FOXO1 and FOXO3 were identified as three targets of miR-182-5p and were regulated by circ_0002483

**Fig. 7:**
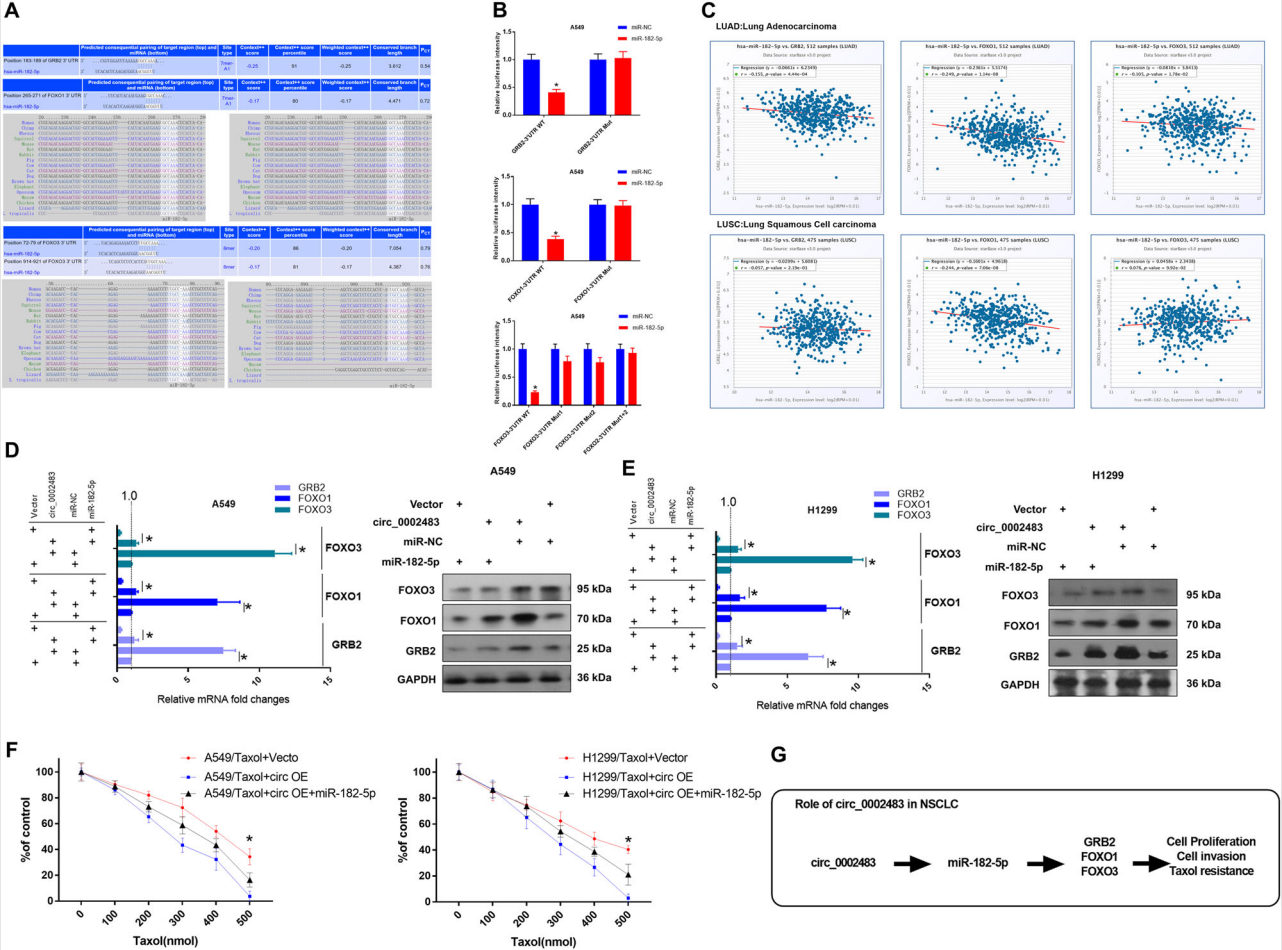
GRB2, FOXO1 and FOXO2 were identified as three targets of miR-182-5p and were regulated by circ_0002483. The interaction between miR-182-5p and GRB2, FOXO1 or FOXO2 was predicted and verified by **a** bioinformatics methods and **b** dual-luciferase reporter assay, *P < 0.05. **c** The correlation between miR-182-5p and GRB2, FOXO1 or FOXO2 in NSCLC tissues. **d**, **e** RT-qPCR and Westerb Blot analysis of GRB2, FOXO1, and FOXO2 in A549 and H1299 cells transfected with miR-182-5p, circ_0002483, or miR-182-5p + circ_0002483, *P < 0.05. **f** A549/Taxol and H1299/Taxol cells transfected with vector, Circ OE or co-overexpressing miR-182-5p, then the cells were analyzed by CCK-8 assay following a 48-h treatment with Taxol (0, 100, 200, 300, 400, or 500 nM), *P < 0.05. **g** A diagram of the molecular mechanisms underlying the circ_0002483/miR-182-5p axis in NSCLC.

Bioinformatics analysis showed that the 3′-UTRs of GRB2, FOXO1, and FOXO2 contained miR-182-5p complementary sequences, which were conserved among species (Fig. 7a). A dual-luciferase reporter assay indicated that miR-182-5p could directly target GRB2, FOXO1, and FOXO2 (Fig. 7b). Gene expression in TCGA Data Portal from starBase v3.0 revealed that GRB2, FOXO1, or FOXO3 expression was negatively correlated with miR-182-5p in LUAD and LUSC tissues (Fig. 7c). In addition, we found that overexpression of circ_0002483 in A549 and H1299 cells led to remarkably upregulated expression of GRB2, FOXO1 and FOXO3, while overexpression of miR-182-5p in A549 and H1299 cells resulted in the opposite effect on GRB2, FOXO1 and FOXO3 expression (Fig. 7d, e). Moreover, our findings suggested that A549 and H1299 cells cotransfected with miR-182-5p and circ_0002483 could restore the expression of GRB2, FOXO1 and FOXO3 back to normal levels (Fig. 7d, e). Furthermore, co-overexpressing miR-182-5p and circ_0002483 rescues the resistance to Taxol in lung cancer cells, indicated circ_0002483 enhances the sensitivity of NSCLC cells to Taxol by sponging miR-182-5p (Fig. 7f). These results suggested that circ_0002483 inhibited the progression and enhanced the Taxol sensitivity of NSCLC through the miR-182-5p/GRB2/FOXO1/FOXO3 signaling pathway (Fig. 7g).

## Discussion

Taxol, which is extracted from the bark of the Pacific Yew tree, is one of the most commonly used anti-tumor agents^21^. Since its approval by the Food and Drug Administration of the United States in 1992 for the therapy of ovarian cancer, Taxol has been widely applied over decades to the clinical therapy for various human cancers^21^. Previous studies have shown that Taxol might exert its anti-tumor activity by disrupting microtubules, which are tube-shaped polymers that play a crucial role in the maintenance of the cell skeleton, in vesicle transport and in cell division^22^. Among all chemotherapeutic drugs, Taxol has shown great efficacy in treating multiple human cancers, such as ovarian cancer, breast cancer and lung cancer^23–25^. At present, Taxol has also become a promising chemotherapeutic agent in the treatment of advanced NSCLC^26,27^. However, most advanced NSCLC patients respond to Taxol treatment only in the initial stage, and acquired Taxol drug resistance frequently occurs in the late period of treatment, resulting in Taxol showing no obvious improvement on the 5-year survival rate of NSCLC patients^27,28^. Therefore, acquired drug resistance to Taxol is no doubt the biggest obstacle to improving the overall response and survival of NSCLC patients.

Although the molecular mechanisms underlying Taxol resistance have not yet been fully elucidated, emerging evidence has shown that miRNAs and lncRNAs play a critical role in the Taxol resistance of NSCLC^29,30^. The roles of circRNAs in the Taxol resistance of NSCLC have barely been investigated. Recently, a circRNA expression profile was reported by Xu Ning et al. using high-throughput circRNA microarrays in parental A549 cells and Taxol-resistant A549/Taxol cells, showing 2909 significantly upregulated and 8372 downregulated circRNAs in the A549/Taxol cells compared with the A549 cells^20^. To further investigate the biological functions of circRNAs in the Taxol resistance of NSCLC, we identified a novel circRNA, circ_0002483, in the top 20 downregulated circRNAs reported by Xu Ning et al. We demonstrated that circ_0002483 inhibited the progression and enhanced the Taxol sensitivity of NSCLC. Mechanistically, five miRNAs (miR-182-5p, miR-520q-3p, miR-582-3p, miR-587, and miR-655) were identified as target miRNAs of circ_0002483, and GRB2, FOXO1 and FOXO3 were verified as target genes of miR-182-5p. Moreover, increased miR-182-5p was demonstrated to act as an oncogene in NSCLC, and circ_0002483 could regulate its target genes (GRB2, FOXO1, and FOXO3) through sponging miR-182-5p.

In conclusion, our findings suggested that circ_0002483 inhibited the progression and enhanced the Taxol sensitivity of NSCLC through the miR-182-5p/GRB2/FOXO1/FOXO3 signaling pathway, providing several potential therapeutic targets to overcome the Taxol resistance of NSCLC. In the future, studies are also needed to explore whether increased expression of miR-182-5p affects Taxol resistance in parental A549 and H1299 cells.

## References

[1] Siegel RL, Miller KD, Jemal A (2019). Cancer statistics, 2019. CA Cancer J. Clin..

[2] Qin H (2018). New advances in immunotherapy for non-small cell lung cancer. Am. J. Transl. Res..

[3] Szejniuk Weronika Maria, Robles Ana I., McCulloch Tine, Falkmer Ursula Gerda Inge, Røe Oluf Dimitri (2018). Epigenetic predictive biomarkers for response or outcome to platinum-based chemotherapy in non-small cell lung cancer, current state-of-art. The Pharmacogenomics Journal.

[4] Lovly CM, Carbone DP (2011). Lung cancer in 2010: one size does not fit all. Nat. Rev. Clin. Oncol..

[5] Goldstraw P (2016). The IASLC lung cancer staging project: proposals for revision of the TNM stage groupings in the forthcoming (eighth) edition of the TNM classification for lung cancer. J. Thorac. Oncol..

[6] Seve P, Dumontet C (2005). Chemoresistance in non-small cell lung cancer. Curr. Med Chem. Anticancer Agents.

[7] Seve P, Reiman T, Dumontet C (2010). The role of betaIII tubulin in predicting chemoresistance in non-small cell lung cancer. Lung Cancer.

[8] Buyukcelik A, Yalcin B, Utkan G (2004). Multidisciplinary management of lung cancer. N. Engl. J. Med..

[9] Zarogoulidis K (2013). Treatment of non-small cell lung cancer (NSCLC). J. Thorac. Dis..

[10] Zhang D, Qiu L, Jin X, Guo Z, Guo C (2009). Nuclear factor-kappaB inhibition by parthenolide potentiates the efficacy of Taxol in non-small cell lung cancer in vitro and in vivo. Mol. Cancer Res..

[11] Li DD (2019). Daurinoline suppressed the migration and invasion of chemo-resistant human non-small cell lung cancer cells by reversing EMT and Notch-1 and sensitized the cells to Taxol. Environ. Toxicol. Pharm..

[12] Chen Y (2016). Pregnane X receptors regulate CYP2C8 and P-glycoprotein to impact on the resistance of NSCLC cells to Taxol. Cancer Med..

[13] Ohta S (1994). Characterization of a taxol-resistant human small-cell lung cancer cell line. Jpn J. Cancer Res..

[14] Yang Z (2019). Noncoding RNA activated by DNA damage (NORAD): biologic function and mechanisms in human cancers. Clin. Chim. Acta..

[15] Ferlita Alessandro La, Battaglia Rosalia, Andronico Francesca, Caruso Salvatore, Cianci Antonio, Purrello Michele, Pietro Cinzia Di (2018). Non-Coding RNAs in Endometrial Physiopathology. International Journal of Molecular Sciences.

[16] Rynkeviciene Ryte, Simiene Julija, Strainiene Egle, Stankevicius Vaidotas, Usinskiene Jurgita, Miseikyte Kaubriene Edita, Meskinyte Ingrida, Cicenas Jonas, Suziedelis Kestutis (2018). Non-Coding RNAs in Glioma. Cancers.

[17] Ors-Kumoglu G, Gulce-Iz S, Biray-Avci C (2019). Therapeutic microRNAs in human cancer. Cytotechnology.

[18] Shang C, Ao CN, Cheong CC, Meng L (2019). Long non-coding RNA CDKN2B antisense RNA 1 gene contributes to paclitaxel resistance in endometrial carcinoma. Front Oncol..

[19] Park GB, Kim D (2019). MicroRNA-503-5p inhibits the CD97-mediated JAK2/STAT3 pathway in metastatic or paclitaxel-resistant ovarian cancer cells. Neoplasia.

[20] Xu N (2018). Profiles and bioinformatics analysis of differentially expressed circrnas in taxol-resistant non-small cell lung cancer cells. Cell Physiol. Biochem..

[21] Weaver BA (2014). How Taxol/paclitaxel kills cancer cells. Mol. Biol. Cell.

[22] Ojima I, Lichtenthal B, Lee S, Wang C, Wang X (2016). Taxane anticancer agents: a patent perspective. Expert Opin. Ther. Pat..

[23] Yamamoto M (2019). AS602801 sensitizes ovarian cancer stem cells to paclitaxel by down-regulating MDR1. Anticancer Res..

[24] Camp Nicola J., Madsen Michael J., Herranz Jesús, Rodríguez-Lescure Álvaro, Ruiz Amparo, Martín Miguel, Bernard Philip S. (2019). Re-interpretation of PAM50 gene expression as quantitative tumor dimensions shows utility for clinical trials: application to prognosis and response to paclitaxel in breast cancer. Breast Cancer Research and Treatment.

[25] Peng C (2014). Effect of Smac and Taxol on non-small-cell lung cancer. Acta Biochim Biophys. Sin. (Shanghai).

[26] Villaruz LC, Socinski MA (2016). Is there a role of nab-paclitaxel in the treatment of advanced non-small cell lung cancer? The data suggest yes. Eur. J. Cancer.

[27] Komuro M, Kaneko M, Narukawa M (2015). Investigation of prognostic factors affecting efficacy in carboplatin- and paclitaxel-based first-line chemotherapies for advanced non-small-cell lung cancer. Tumori.

[28] Yen WC (2004). A selective retinoid X receptor agonist bexarotene (Targretin) prevents and overcomes acquired paclitaxel (Taxol) resistance in human non-small cell lung cancer. Clin. Cancer Res..

[29] Wang P, Chen D, Ma H, Li Y (2017). LncRNA SNHG12 contributes to multidrug resistance through activating the MAPK/Slug pathway by sponging miR-181a in non-small cell lung cancer. Oncotarget.

[30] Peng B (2018). Knockdown of miR935 increases paclitaxel sensitivity via regulation of SOX7 in nonsmallcell lung cancer. Mol. Med. Rep..

